# Study of the Effect of Cell Prestress on the Cell Membrane Penetration Behavior by Atomic Force Microscopy

**DOI:** 10.3390/mi14020397

**Published:** 2023-02-05

**Authors:** Guocheng Zhang, Yufang Chang, Na Fan, Bin Yan, Xianmeng Li, Zihan Yang, Zhenyang Yu

**Affiliations:** 1 Department of Mechanical Engineering, Anyang Institute of Technology, Yellow River Avenue West, Anyang 455000, China; 2 School of Accountancy, Anyang Institute of Technology, Yellow River Avenue West, Anyang 455000, China; 3 School of Mechanical and Electrical Engineering, University of Electronic Science and Technology of China, 2006 Xiyuan Avenue, Hi-Tech West District, Chengdu 611731, China

**Keywords:** atomic force microscopy, insertion rate, stiffness, prestress, substrate strains

## Abstract

In recent years, atomic force microscopes have been used for cell transfection because of their high-precision micro-indentation mode; however, the insertion efficiency of the tip of AFM into cells is extremely low. In this study, NIH3T3 mouse fibroblast cells cultured on a flexible dish with micro-groove patterns were subjected to various substrate strains at 5%, 10%, 15%, and 20%. It was found that the cell stiffness depends on the prestress of the cell membrane, and that the insertion rate of AFM tips into the cell membrane is proportional to the stiffness through the AFM indentation experiment. The finite element analysis proves that prestress increases the bending stiffness of the cytoskeleton, allowing it to better support the cell membrane, which realizes the stress concentration in the contact area between the AFM tip and the cell membrane. The results indicate that the prestress contributes to the mechanical properties of the cell and suggest that the insertion efficiency could be greatly improved with an increase of the prestress of the cell membrane.

## 1. Introduction

The technique of precisely transferring biological agents, such as DNA or RNA, into a single cell can accelerate the development of gene therapy drugs [1]. There are various methods of introducing foreign DNA into the eukaryotic cell: some rely on chemical materials (calcium phosphate, liposomes, and kinds of cationic materials) [2,3,4], some on physical treatment (bulk electroporation, microinjection) [5,6], others on biological particles (viruses) [7]. However, the targeted cells are randomly transfected, and these traditional methods cannot obtain the dosage controllability of the transfection agent.

With the development of molecular biology tools, AFM has exhibited promising potential for visualizing and manipulating biological samples with nanometer resolution under near-physiological conditions [8,9,10]. The ability to maintain accurate spatial and temporal control of probes such as decorated AFM tips with force feedback has made it possible to use them as delivery tools for application in cell transfection, which minimizes cell damage [11,12]. Michel Grandbois et al. [11] modified plasmid DNA encoding for fluorescent protein EGFP on a commercial silicon nitride tip of AFM and successfully transfected human embryonic kidney cells (HEK 293) without inducing critical cell damage. Their successful transfection rate was only 30%. The cell transfection efficiency depends on the insertion rate of the AFM tip piercing the cell membrane. Our group found that the insertion rate is very dependent on the shape. Etched silicon nitride tips 200 nm in diameter have achieved a 70% penetration rate of the cell membrane, which was much higher than that of unprocessed commercial tips [13]. Although a high penetration rate can be achieved by using fine nano-needle tips, there is a potential application problem, that is, how to carry drugs with such fine needles. Additionally, this method does not guarantee that the DNA molecules attached to the AFM tip will release only in the cytoplasm and not diffuse in the cell culture medium. To solve this problem, Zambelli et al. and Espinosa et al. developed fluidic force microscopy (FluidFM), which combines the nanoscale force-controlled positioning of AFM, and a pressure-controlled fluid delivery through an integrated micro-channel [14,15]. Zambelli et al. used this FluidFM system for straightforward and standardized injection into cell nuclei and obtained a 40% transfection efficiency [14]. However, the statistical transfection efficiency was based on the condition that cell membranes were punctured, ignoring those cells that are not punctured. Limited by the current micromachining technology, only large AFM probes can be etched out of the microchannel. According to our previous experimental experience, large AFM probes have been shown to have difficulty in piercing the cell membrane [13]. Therefore, though the FluidFM solves the problems of dosage control and precise localization of transfection, the insertion rate of the AFM probe piercing the cell membrane is restricted by the large size of the FluidFM tip.

According to many other studies, the stiffness depends on the prestress and is based on strain hardening regimes [16,17]. Here, the insertion rate of large commercial AFM tips into NIH3T3 cells was investigated at different stiffness conditions that resulted from prestress to the cells. The results of our experiments and simulations are of great significance to the improvement of cell transfection efficiency through AFM with large tip sizes.

## 2. Materials and Methods

### 2.1. PDMS Culture Dish Preparation

The cell culture dish is made of two pieces of silicone elastomer (polydimethylsiloxane—PDMS) bonded together in the plasma cleaner chamber. A hole was dug in the center of the top piece of PDMS, and the bottom piece of PDMS was imprinted with micro-groove patterns. The micro-groove patterns were formed by soft photolithography and the preparation process is shown in Figure S1.

### 2.2. Cell Culture

The NIH3T3 cells (ATCC) were cultured in high glucose Dulbecco’s Modified Eagle’s Medium (Gibco, Thermo Fisher Scientific, Waltham, MA, USA) and supplemented with 10% bovine calf serum (*v*/*v*, Gibco, Thermo Fisher Scientific, Waltham, MA, USA) in a Petri dish (35 mm in diameter) at 37 °C under 5% CO_2_ atmosphere until they were subconfluent. Then, 1 mL Trypsin-EDTA solution (Gibco, Invitrogen, Carlsbad, CA, USA) was added to the Petri dish, and the collected cells were then passaged and seeded in PDMS culture dish.

### 2.3. Cell Stretching

The prestress of the cells was achieved by stretching the substrate. Both sides of the PDMS culture dish are clipped by two clamps. The PDMS dish is stretched by the motor controlled by the microprocessor. The stretching device is shown in Figure S2. The stretching speed is set about 2.5 mm/min. Cell images were acquired under an inverted microscope.

### 2.4. Cell Stiffness Measurement and Cell Insertion Behavior

The cell stiffness measurement and cell insertion behavior were both conducted by AFM indentation experiment under force–distance mode. The difference between them is that the stiffness measurement was assessed using a spherical glass tip (diameter: 4.5 µm) and the insertion behavior was studied using a traditional pyramidal silicon nitride tip (Tip Radius:10 nm, MSCT, Bruker, NY, USA). The spring constants of the spherical tip and pyramidal tip were 0.05 N/m and 0.02 N/m, respectively. An AFM force–displacement curve describes the relationship of tip-sample interaction forces vs. tip-sample distance. Before the indentation, a “trigger force” was set, which means that the AFM tip will retract in the indentation process when the cell is subjected to a force equal to it. The stiffness of the cell is expressed by the elastic modulus. The elastic modulus was obtained by establishing the contact model between the tip and the sample based on the data of the force–displacement curve in the AFM indentation experiment. For the spherical tip, the Hertz model is the most commonly used method to measure the elastic modulus [18]. The elastic modulus of the cell *E* was calculated by the following equation:

$$F=\frac{4\sqrt{R}E}{3\left(1-v^{2}\right)}\delta^{\frac{3}{2}}$$

where *F* is the normal force, *v* is Poisson’s ratio (about 0.5 used in cell [19]), and *δ* is the tip indentation depth. It is worth noting that the indentation depth here is not the displacement of the Piezo actuator, but the value obtained by subtracting the displacement of the cantilever deflection. The cantilever deflection can be obtained by *d* = *F*/k, k is the spring constant of cantilever.

If the cell membrane is penetrated by the AFM tip, a “force drop” would be shown in the force–displacement curve in the approach process, with the corresponding force being defined as the “insertion force”. All the measurements were completed in a very short period of time by an AFM (Agilent 5500, Agilent Technologies, Santa Clara, CA, USA) in contact mode.

### 2.5. Actin and Nucleic Acid Staining

DNA and actin were stained with the standard DAPI (Thermo Fisher Scientific, Waltham, MA, USA) and FITC-Phalloidin (P5282, Sigma), respectively. The FITC-Phalloidin and DAPI were diluted to 5 and 50 µg mL^−1^ by staining buffer, respectively. The cells were fixed in 4% paraformaldehyde (Sigma-Aldrich) for 15 min in a refrigerator at 4 °C and permeabilized with 0.1% Triton X-100, and 1% Bovine Serum Albumin (BSA) (Sigma-Aldrich). Then, cells were incubated with the FITC-Phalloidin solution in the dark at 37 °C for 60 min. After that, the staining solution was removed, and the stained cells were further rinsed with PBS, twice. Then, the stained cells were again incubated with the DAPI solution for 10 min and rinsed with PBS, twice. Finally, the immunofluorescence images were collected by the confocal microscope (Leica TCS SP8, Germany) using 408 and 488 nm filters for DAPI and FITC, respectively.

### 2.6. Finite Elements Analysis

To investigate the effect of the prestress on the stiffness of the cell, a numerical model composed of the cell membrane, cross-linked actin filaments network, and stress fibers were established. The diameter of the actin filament is approximately 7 nm, and the stress fibers are usually composed of 10–30 actin filaments [20]. The cytoskeleton consists of a square plane of orthogonal, equally spaced, cross-linked arrays of individual filaments and stress fibers. The network topology is designed to mimic orthogonal networks such as the actin cortex [21]. Here, the actin filament of 38.46 nm^2^ in the sectional area and the actin filaments of 400 nm^2^ in sectional area were linked into the network in this model. The cell membrane was set as 5 nm in thickness. The dynamic calculation method of ABAQUS/Explicit was used to simulate the indentation process. Both actin filaments and actin filaments bundles were set as beam elements, which can be used to simulate the slim, line-like structures for which the section size of the beam is small enough to allow comparison with the typical size of the beam axial direction. The cell membrane was set as a membrane element, which are used to represent a thin surface in space that provides strength but no bending stiffness in the plane of the element. The detailed material properties of all the components used in our FE model are given in our previous study [13]. ”Tie” constraint was set at the cell membrane–actin filaments, cell membrane–actin bundles, and actin filaments–actin filaments bundles, meaning that there was no relative motion at their junction.

## 3. Results and Discussion

### 3.1. Cell Alignment

**Figure 1:**
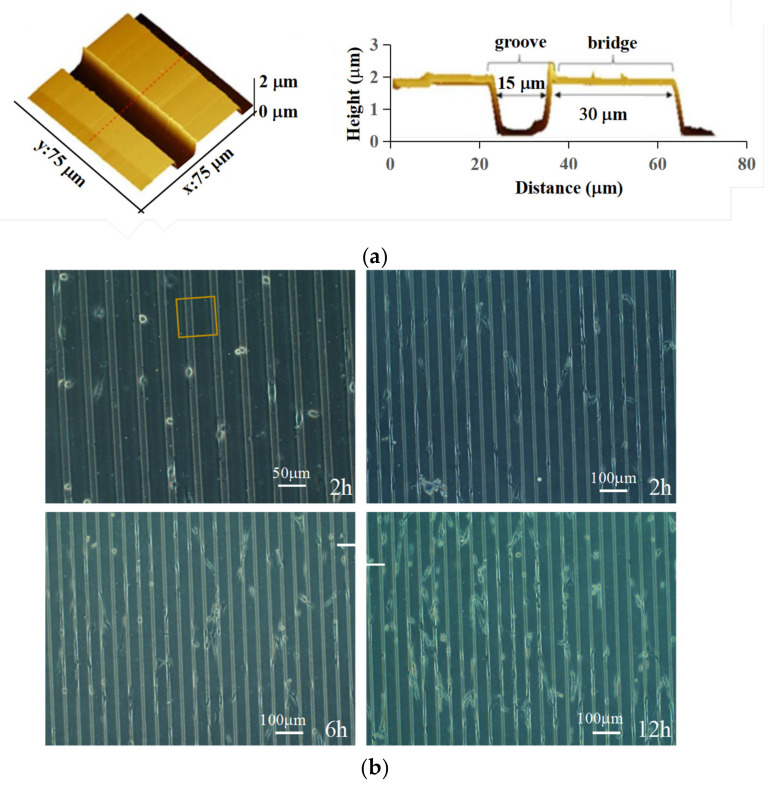
(**a**) AFM morphology of patterned substrate; (**b**) cells spread on the flexible culture dish with micro-patterns for 2 h, 4 h, 6 h and 12 h.

The effects of the micro-pattern in a culture dish on cell alignment have been reported in many studies [22,23]. PDMS has been used to prepare micro-patterns in culture dishes due to its excellent combination of biocompatibility and flexible properties. Here, the size effect of the micro-pattern was investigated on the alignment of cells and it was found that micro-patterns with width between 15 μm and 30 μm allowed cells to grow more effectively along the pattern, while the growth direction of cells was random on the micro-patterns with smaller or larger width sizes. The growth of cells at different channel sizes is shown in Figure S3. In this study, the culture dish with a microgroove of 15 µm in width and a bridge of 30 μm in width was chosen to culture the cells. Figure 1a shows the AFM morphology of the yellow rectangular region in Figure 1b. The results show that the cells align well in the microgrooves of 15 µm. The growth process of the cells was recorded after seeding for 12 h, as shown in Figure 1b. The cells aligned well in 4 h, and the cell alignment phenomenon occurred not only in the groove but also on the bridge. Therefore, a flexible substrate with microgrooves of 15 µm in width was used as the cell culture dish in order to better unify the cell prestress.

### 3.2. Apply Prestress to The Cells

The prestress was applied to the cells by stretching the substrate with microgrooves of 15 um. To verify that the stretching of the PDMS culture dish had been transferred to the cell, the changes of cell shape were tracked under an inverted microscope.

**Figure 2:**
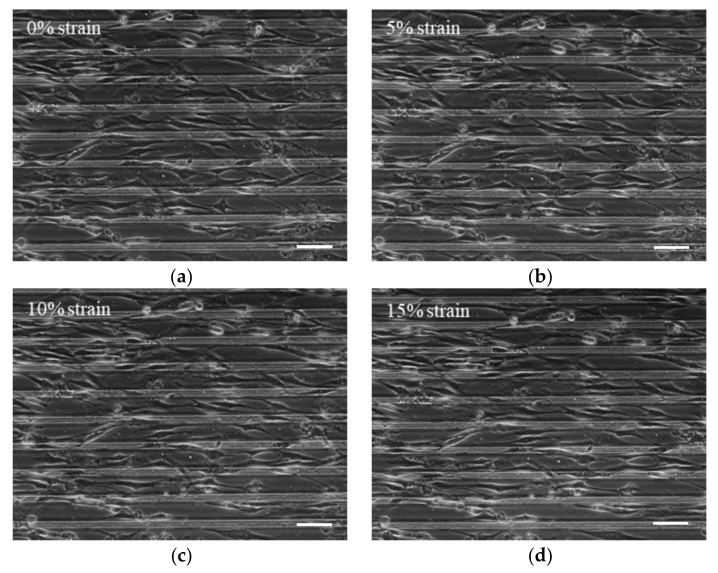

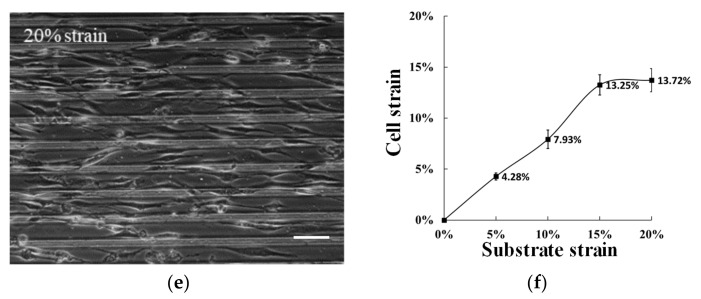
Cell stretching in PDMS culture dish with micro-patterns. The cells were stretched at 0% (**a**), 5% (**b**),10% (**c**), 15% (**d**), and 20% (**e**) strains of the substrate. (**f**) The transmission of PDMS culture dish stretching to the cells (scale bar = 50 µm).

NIH3T3 cells were subjected to various strains in the stretching device. As soon as the stretching ratio reached the set value, the state of the cells was recorded immediately. A clear elongation of culture dish along the stretch direction was detected in Figure 2. The transmission from PDMS culture dish to cell membrane depends on the tightness of cell adherence to the collagen-coated PDMS culture dish. Ten cells were chosen to describe the relationship between PDMS strain and cell strain. The strain along the longitudinal direction can be calculated as

(1) $$\varepsilon =(L_{1}-L_{0})/L_{0}$$

where *L*_0_ and *L*_1_ are the lengths of cells or substate before and after stretching, respectively. The stretching ratio of the cell increases with the increment of the stretching rate of the PDMS culture dish, as shown in Figure 2f. While the strains of PDMS are 5%, 10%, 15%, and 20%, the corresponding strains of the cells are 4.28%, 7.93%, 13.25%, 13.72%, respectively. According to Kozaburo’s report [24], fibroblast cells can be stretched up to four times the original length without breaking. Therefore, the cellular activity cannot be impaired when they are stretched by 13.72%. This assumption is confirmed from the subsequent culture after stretching. The cell stretch ratio is slightly smaller than the stretch ratio of the PDMS culture dish when the PDMS strain does not exceed 15%. However, compared with the cell strain when PDMS stretching ratio is 15%, the cell strain does not increase significantly when the PDMS stretching ratio reaches 20%. This suggests that there is an interfacial slipping between the cell and the substrate. This is mainly because large strains, such as focal adhesions, disrupt the cell–matrix junction.

### 3.3. Cell Stiffness

#### 3.3.1. Measurements of Cell Stiffness

**Figure 3:**
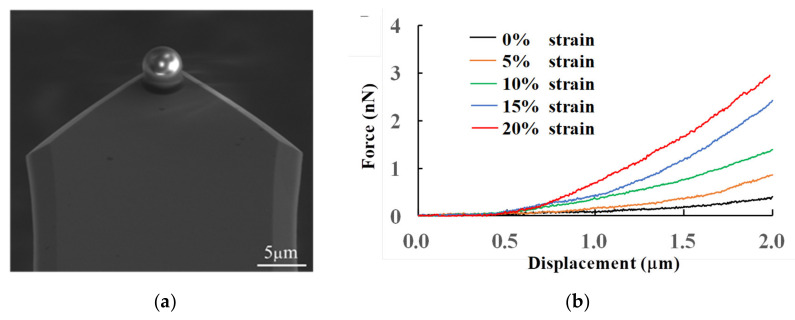
The force–displacement curves under the indentation depth of 1.5 µm when using the spherical glass tip. (**a**) An SEM image of the spherical glass tip. (**b**) The representative force–displacement curves under different substrate strains.

The spherical tip of the AFM probe was used to measure the stiffness of cells. The morphology of the tip is characterized by scanning electron microscope (SEM), as shown in Figure 3a. The large contact area between the spherical tip and the cell prevents damage to the cell even when the tip presses deeply into the cell, thus enabling the detection of the mechanical properties of its entire structure. The representative force–displacement curves at different strain states are plotted in Figure 3b. The average values of the stiffnesses were calculated, by Hertz fitting, to be 423 ± 72 Pa, 608 ± 43 Pa, 1321 ± 26 Pa, 1710 ± 54 Pa, and 1862 ± 69 Pa, which correspond to the respective PDMS strains of 0%, 5%, 10%, 15%, and 20%. The results show that the stiffness of the NIH3T3 cell increases with the increasing stretch ratio. However, when the strain attained 20%, the stiffness increased in a way that is not obviously compared with the substrate strain of 15%. All tests were completed in a very short period of time due to the way in which the strain and stress of the actin cross-linking network is dependent on time [17].

**Figure 4:**
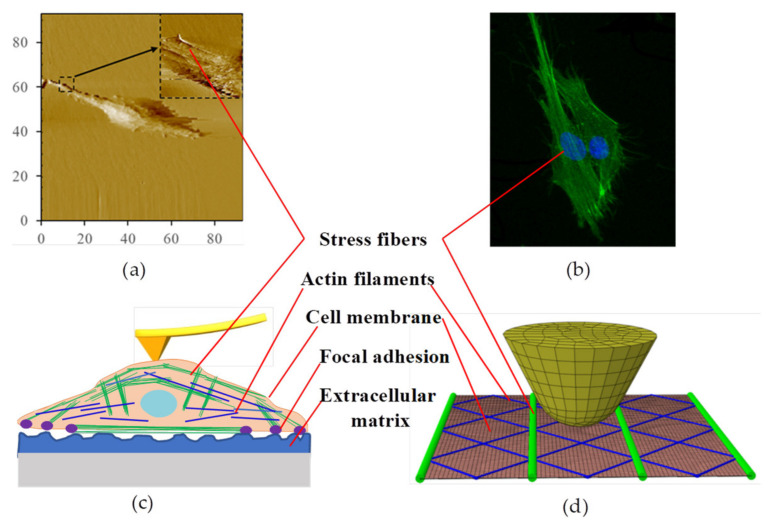
The finite element analysis model and simulation result. (**a**) AFM topography image of a single NIH3T3 cell. (**b**) Immunofluorescence image of NIH3T3 cells. (**c**) The schematic of a cell adhering to the substrate. (**d**) The finite element analysis model.

To explain the increased stiffness of the cells by stretching the substrate, the cytoskeleton—which dominates the mechanical properties of the cell—needs to be introduced. It is known that the cytoskeleton is essential in maintaining cell shape, movement, and anchorage [25]. The cytoskeleton is composed of actin filaments—which play a major role in cell mechanical properties—intermediate filaments, and microtubules. The actin filament network is located below the cellular cortex, which supports and strengthens the cell membrane. This network allows cells to maintain their shapes. Additionally, the actin filaments together with myosin and other actin-binding proteins often form bundles known as actin stress fibers. F-actin shows a faster-than-linear strain hardening behavior [21]. Many of the stress fibers are arranged parallel to the long axis of the cell. The stress fibers are seen along the long axis of the cell in the AFM image, as shown in Figure 4a and which can also be confirmed from the immunofluorescent image in Figure 4b. These conduct force by connecting to the extracellular matrix through the focal adhesions on the cell membrane [26]. Therefore, when the substrate is stretched, both the cell membrane and the stress fibers are prestressed by the focus adhesion and the complex actin filament network.

Many researchers have studied the skeleton of cells according to the beam theory [27,28], therefore, the bending equation of the beam could be written as:

(2) $$M(x)=\frac{F_{2}}{2}x-F_{1}w$$

where *M(x)* is bending moment, *F*_1_ is tension, *F*_2_ is pressure, and *w* is the deflection. Therefore, when stretching the substrate, the axial tension actually reduces the value of the bending moment of the stress fibers. The reduction of the bending moment improves the bending rigidity of the skeleton. Therefore, when the AFM tip indents the cell, the cell membrane is not easily collapsed because it is supported by the large bending rigidity of the cell skeleton, this in turn means it is more likely to cause a concentration of stress and to thereby penetrate the cell membrane.

#### 3.3.2. Finite Element Analysis

**Figure 5:**
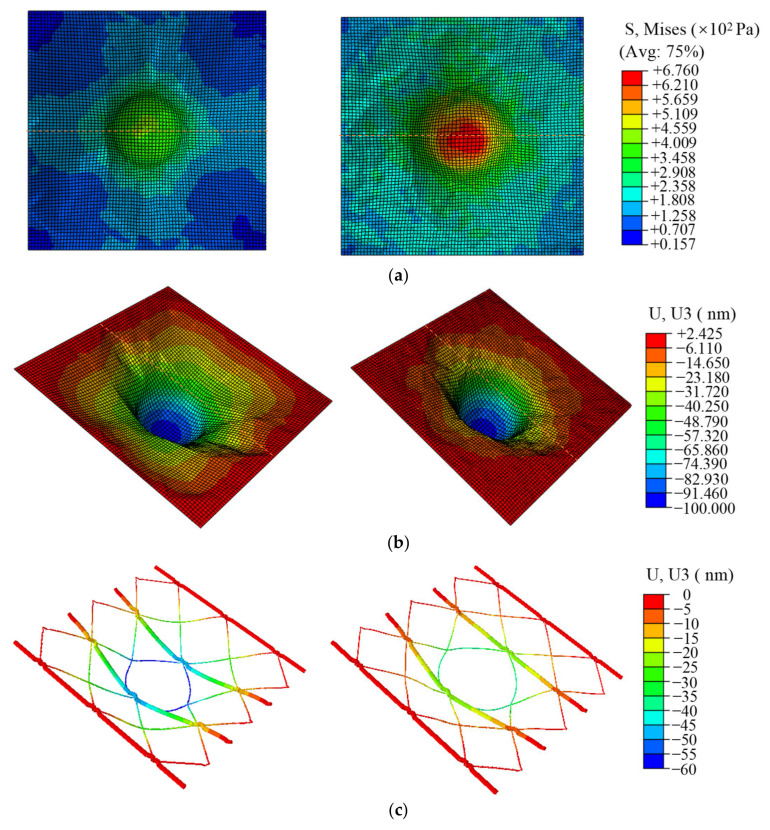
(**a**) The stress distribution of the cell membrane under the indentation depth of 100 nm in the absence (**left**) and presence (**right**) of prestressing. (**b**) The deformation of the cell membrane in the z-direction under the indentation depth of 100 nm in the absence (**left**) and presence (**right**) of prestressing. (**c**) The deformation of the cell actin filaments in the z-direction under the indentation depth of 100 nm in the absence (**left**) and presence (**right**) of prestressing.

In order to better understand the effect of cell prestress on cell stiffness, a finite element analysis was performed. The finite element analysis model was composed of AFM tip, cell membrane, and actin filaments network, as well as stress fibers composed of microfilaments, as shown in Figure 4d. Two kinds of boundaries were set in our model: the prestresses of 100 Pa, 50 kPa, and 50 kPa were applied on the membrane, actin filaments, and stress fibers respectively for one case, while for another case no prestress was applied. The conical tip with a curvature radius of 50 nm was built on the top of the membrane, and was moved 100 nm toward the cell membrane in the simulation. The stress distribution on the cell membrane and the displacement of the cell membrane and cytoskeleton along the indentation direction are obtained when the tip moves 100 nm down in the absence of prestressing and also in the presence of prestressing (Figure 5). It can be seen from the simulation results that the stress on the cell membrane with prestress is higher than that without prestress. In the prestressed case, the maximum stress on the cell membrane is 676 Pa, while in the unprestressed case, the maximum stress on the cell membrane is 410 Pa. Even after subtracting the initial stress of 100 Pa on the cell membrane, the stress of the cell membrane with prestress is about 160 Pa larger than that without prestress. It was found that the prestressed cytoskeleton is less bent than the unprestressed cytoskeleton during the process of tip indentation. This result is consistent with the bending theory of the beam under axial force as discussed above. Therefore, the prestressed cell membrane supported by the cytoskeleton in the area around the tip does not collapses less than that of the unprestressed membrane.

**Figure 6:**
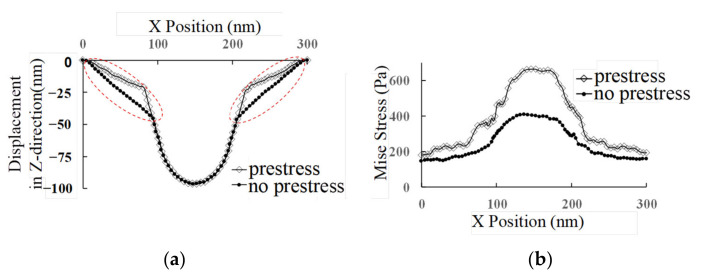
Displacement and stress distribution of the cell membrane in the z-direction along the *x*-axis midline with and without prestress. (**a**) Displacement of the cell membrane in the z-direction. (**b**) Stress distribution of the cell membrane.

The z-directional displacement and stress distribution of the cell membrane from the midline position (orange dashed line shown in Figure 5b) of the cell membrane were extracted, as shown in Figure 6. It can be seen from the image that the displacement of the cell membrane with the tip contacting the cell membrane region downward is significantly smaller in the case with prestress than in the case without prestress. Similarly, in the case with prestress, the stress at all locations of the cell membrane is greater than that of the cell membrane without prestress. This implies that the stress threshold for cell membrane penetration in the contact region is reached more quickly when the cell is under higher prestress. The region of stress peak with prestress is narrower than that without prestress, which demonstrates that after applying prestress to the cell, the indentation process of AFM is more likely to achieve stress concentration on the cell membrane surface and therefore also more likely to result in cell membrane puncture.

### 3.4. Insertion Rate

**Figure 7:**
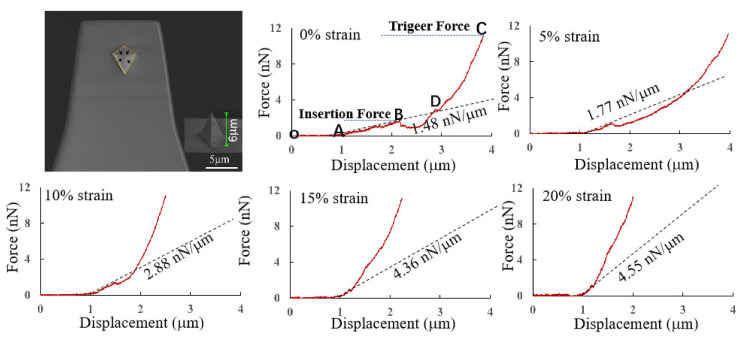
The SEM image of the pyramidal silicon nitride tip and the representative force–displacement curves under different substrate strains. (O is the position where the tip starts to move downward; A is the position where the tip starts to touch the cell; B is the position where the tip pierces the cell; C is the position where the force between the tip and the cell reaches the set maximum force, and D is the position where the tip pierces the nucleus).

The penetration behavior occurs when the value of tensile stress on the puncture point exceeds the threshold value of damage stress. Based on our previous research, the optimal penetration behavior depends on two conditions: one is the rapid growth of stress before stress propagation and the other is increased stress on the cell membrane surface [13]. From the experimental and simulation results discussed above, the method of stretching the culture substrate of the cells can effectively satisfy these two conditions. To verify that our approach can improve the insertion rate of AFM tips into cell membranes, indentation experiments were performed by using the pyramid AFM tip, as shown in Figure 7. Here, the trigger force was set at 12 nN, and the speed of the AFM tip was set at 1 μm/s for our experiments. The indentation manipulation was repeated three times on 20 cells on each experiment. Thus, 60 force–displacement curves were obtained for each prestress condition. The representative force–displacement curves are shown in Figure 7. The schematic diagram of the AFM tip penetrating into the cell is shown in Figure S4 of the supplementary material, as can be seen in Figure 7. The experimental process of puncturing the cells is divided into four stages: from non-contact to contact (O–A), pressing in (A–B), puncturing (B), and continuing to press in (B–C). In the O–A stage, the tip had no contact with the cells, and the force was 0. After reaching the A contact point, the force began to increase. Because the cellular cortex is generally regarded as an elastic material, the force–displacement curve in the A–B stage can be considered to be linear, and the force clearly decreases after reaching the B point, which is also the mark of cell penetration. After puncture, the tip enters the cytoplasm. The cytoplasm is considered to be a viscoelastic material, and the force–displacement curve is no longer linear. With continuous pressing, the force increases significantly faster, due to the result of the substrate effect or contact with the nucleus. Two force drops can be observed in the force–displacement curve without strain in Figure 7, which means that there are two puncture behaviors during the tip downward pressure. At point A, the tip is pressing into the cell membrane and the nucleus is penetrated at point B. The subsequent force increases faster. In our other studies [8], the height of the cell is generally 3–5 mm, so the reaction force between the tip and the cell increases sharply in the later stage of displacement because it may come into contact with a hard substrate.

The detection of mechanical properties of cells using AFM is mainly influenced by the measurement parameters (tip shape and loading rate) and the detection location [8,18]. While the cell is highly heterogeneous, the stiffness of the nucleus and cytoplasm regions differs greatly, and the large contact area between the pyramid AFM tip and the cells greatly increases the errors caused by heterogeneity. It was also found that the Young’s modulus of cells obtained with the spherical tip was substantially higher than that of the spherical tip [18]. Moreover, the cell membrane is a linear elastic material, so the curve from the contact point A to the puncture point B is simply linearized here. The dashed line in Figure 7 is obtained by linearly fitting the curve from the initial contact between the tip and membrane to the cell membrane penetration, as shown in Figure 7. It can be seen that the slope increases as the prestress increases. Higher slope means higher cell stiffness. This result is consistent with the results of cell elastic modulus under different strain states measured by spherical tip above. This means that the stress of the cell membrane damage threshold will be achieved earlier in the contact area when the cells are under higher prestress. Thus, based on our previous research, we can say that it will cause the cell membrane to be penetrated much more easily [10].

The statistical analysis of the insertion behaviors above under different substrate strain states is shown in Table 1. At a fixed downward pressure speed of 1 um/s and a fixed trigger force of 12 nN, the insertion rate of the tip into the cells increased significantly with increasing cell strain. Compared with the 19% insertion rate at 0% stretch ratio, the insertion rate increased to 39%, 52%, 60%, 62% at 5%, 10%, 15%, 20% stretch ratio, respectively. It can be seen that the cell insertion rate at the 15% stretch rate does not increase significantly compared with the cell insertion rate at the 20% stretch rate. This is mainly because the stress fibers may break under large strains and there is a slide between the cells and the substrate. This remains consistent with the previously measurement results of strain transfer from substrate to cells and stiffness of cells being not significantly different at these two tensile states. Additionally, the results show that the average insertion force and displacement decrease with increasing substrate strain. Cell membranes under prestress are more likely to concentrate stress, allowing it to reach the stress threshold for mechanical damage to the cell membrane more quickly in the contact region during the indentation process. Thus, smaller forces are required to achieve the cell membrane’s threshold of stress and penetrate the cell. The result is consistent with finite element simulation. Moreover, in our previous study [13], the stress transfer of the cell membrane can be divided into two stages during the indentation process. In the first stage, the stress of the cell membrane increases rapidly, while the stress of the actin filaments increases slowly. In contrast, the stress of the actin filaments grows dramatically in the second stage. The optimal insertion behavior should occur in the first stage. Briefly, the stress on the surface of the cell membrane does not always increase with an increase of indentation force. The average insertion time and displacement refer to the time and distance between the contact of the tip with the cell (Point A) and the appearance of the puncture behavior (Point B) extracted from the force–displacement curve with abstraction of the bending distance of the cantilever beam. It can be seen that the insertion time decreases with increasing strain, which confirms that the cell penetration behavior is likely to occur in the first stage of stress transfer.

**Figure 8:**
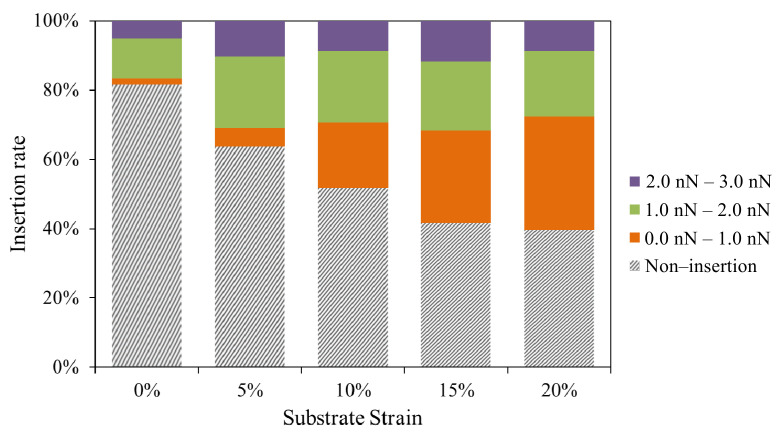
The statistical analysis of the insertion rate under different substrate strains and insertion force distribution.

Figure 8 shows the distribution of the insertion force for the successful occurrence of piercing behavior of the cells under different strains. The data are obtained by analyzing 60 force–displacement curves under each prestress condition. It can be seen that the insertion force distribution is between 0–3 nN for different strain conditions. The difference is that under small strain conditions (0–10%), most of the insertion force is between 1 nN and 2 nN, while under large strain conditions (15–20%), the insertion force ranges from 0 nN to 1 nN. Compared with the average insertion force of 2.0 ± 0.3 nN under no strain conditions, the insertion forces are 1.0 ± 0.2 nN and 1.0 ± 0.3 nN at 15% and 20% strain, respectively. The smaller insertion force does not mean that the failure stress of the cell membrane is smaller, but that the effective contact area between the strained cell membrane and the tip is smaller at the micro level. The membrane molecules in tensile strain have less contact with the tip resulting in the force decreasing, which has been verified in our previous molecular dynamics simulation [10]. Although the trigger forces are all set as 12 nN, the cell penetration rate was only 19% in the strain-free condition. It can be concluded that the cell piercing behavior is independent of the trigger force. Mechanical disruption of the cell membrane is essentially the result of stress at a regional site of the cell membrane reaching the limit of the stress it can withstand. Due to the heterogeneous property of the cell, larger forces do not increase the stress on the cell membrane indefinitely, but transfer the stress to the cytoskeleton that maintains the cell shape and other organelles.

## 4. Conclusions

In summary, we aimed to investigate the insertion mechanism of a living cell from a mechanical point of view, for the purpose of improving insertion efficiency. It is proved that the stiffness of cells can greatly improve their penetration rate. This is of great significance to future cell transmission systems. We increased the prestress of the cells by stretching the cell culture substrate, thus increasing the stiffness of the cell membrane and ultimately improving the penetration rate of the probe into the cell membrane. Through finite element analysis, we found that the cytoskeleton, which dominates the mechanical properties of the cell, allowed the stress to increase more rapidly when the probe pressed down in the presence of prestress. The cell membrane under increased prestress will reach the cell membrane breakage stress threshold more rapidly under smaller forces. The hypothesis of the prestress effect of the cell membrane was numerically and experimentally supported to explain the insertion behavior. In our study, higher penetration rates were achieved by modifying the cell membrane (62%) at 20% stretch ratio in order to increase its stress. Our research work reveals the mechanism of cell membrane penetration, which provides enhanced versatility in the use of AFM, allows application of this technology in other fields, such as transfection, intracellular study, cell diagnosis, and biosensors.

## Figures and Tables

**Table 1:** The statistical analysis of the insertion behaviors under different substrate strain states.

Trigger Force (nN)	Substrate Strain	Average Insertion Force (nN)	Average Insertion Displacement (μm)	Indentation Speed (μm/s)	Average Insertion Time (s)	Insertion Rate
12	0%	2.0 ± 0.3	1.2 ± 0.5	1	1.2 ± 0.5	19% (11/60)
12	5%	1.8 ± 0.3	0.7 ± 0.3	1	0.7 ± 0.3	39% (23/60)
12	10%	1.5 ± 0.2	0.5 ± 0.1	1	0.5 ± 0.1	52% (31/60)
12	15%	1.0 ± 0.2	0.2 ± 0.1	1	0.2 ± 0.1	60% (36/60)
12	20%	1.0 ± 0.3	0.2 ± 0.1	1	0.2 ± 0.1	62% (37/60)

## Data Availability

The data that support the findings of this study are available from the corresponding author, upon reasonable request.

## Supplementary Materials

The following supporting information can be downloaded at: https://www.mdpi.com/article/10.3390/mi14020397/s1, Figure S1: The micro-groove patterns were formed by photolithography; Figure S2: Stretching equipment; Figure S3: The cells grow in micro pattern with groove width of 5 mm (a), 10 mm (b) and 15 mm (c) after seeding for 12 h; Figure S4: The representative Force-Displacement curve of AFM tip penetrating the cell.

## References

[1] Du X., Wang J., Zhou Q., Zhang L., Wang S., Zhang Z., Yao C. (2018). Advanced physical techniques for gene delivery based on membrane perforation. Drug Deliv..

[2] Hamann A., Nguyen A., Pannier A.K. (2019). Nucleic acid delivery to mesenchymal stem cells: A review of nonviral methods and applications. J. Biol. Eng..

[3] Martínez-Negro M., Barrán-Berdón A.L., Aicart-Ramos C., Moyá M.L., de Ilarduya C.T., Aicart E., Junquera E. (2018). Transfection of plasmid DNA by nanocarriers containing a gemini cationic lipid with an aromatic spacer or its monomeric counterpart. Colloids Surf. B Biointerfaces.

[4] Zhang J., Hu Y., Wang X., Liu P., Chen X. (2019). High-Throughput Platform for Efficient Chemical Transfection, Virus Packaging, and Transduction. Micromachines.

[5] Canoy R.J., André F., Shmakova A., Wiels J., Lipinski M., Vassetzky Y., Germini D. (2020). Easy and robust electrotransfection protocol for efficient ectopic gene expression and genome editing in human B cells. Gene Ther..

[6] Fujii M., Matano M., Nanki K., Sato T. (2015). Efficient genetic engineering of human intestinal organoids using electroporation. Nat. Protoc..

[7] Hardee C.L., Arévalo-Soliz L.M., Hornstein B.D., Zechiedrich L. (2017). Advances in Non-Viral DNA Vectors for Gene Therapy. Genes.

[8] Zhang G., Fan N., Lv X., Liu Y., Guo J., Yang L., Peng B., Jiang H. (2017). Investigation of the Mechanical Properties of the Human Osteosarcoma Cell at Different Cell Cycle Stages. Micromachines.

[9] Ke C., Humeniuk M., S-Gracz H., Marszalek P.E. (2007). Direct measurements of base stacking interactions in DNA by single-molecule atomic-force spectroscopy. Phys. Rev. Lett..

[10] Zhang G., Jiang H., Fan N., Yang L., Guo J., Peng B. (2018). Molecular dynamics simulation of cell membrane penetration by atomic force microscopy tip. Mod. Phys. Lett. B.

[11] Cuerrier C.M., Lebel R., Grandbois M. (2007). Single cell transfection using plasmid decorated AFM probes. Biochem. Biophys. Res. Commun..

[12] Meister A., Gabi M., Behr P., Studer P., Vörös J., Niedermann P., Bitterli J., Polesel-Maris J., Liley M., Heinzelmann H. (2009). FluidFM: Combining Atomic Force Microscopy and Nanofluidics in a Universal Liquid Delivery System for Single Cell Applications and Beyond. Nano Lett..

[13] Fan N., Jiang H., Ye Z., Wu G., Kang Y., Wang Q., Ran X., Guo J., Zhang G., Wang G. (2018). The Insertion Mechanism of a Living Cell Determined by the Stress Segmentation Effect of the Cell Membrane during the Tip-Cell Interaction. Small.

[14] Guillaume-Gentil O., Potthoff E., Ossola D., Dörig P., Zambelli T., Vorholt J.A. (2013). Microfluidics: Force-Controlled Fluidic Injection into Single Cell Nuclei (Small 11/2013). Small.

[15] Kang W., Yavari F., Minary-Jolandan M., Giraldo-Vela J.P., Safi A., McNaughton R.L., Parpoil V., Espinosa H.D. (2013). Nanofountain Probe Electroporation (NFP-E) of Single Cells. Nano Lett..

[16] Coughlin M.F., Stamenović D. (2003). A Prestressed Cable Network Model of the Adherent Cell Cytoskeleton. Biophys. J..

[17] Lam R.H.W., Weng S., Lu W., Fu J. (2012). Live-cell subcellular measurement of cell stiffness using a microengineered stretchable micropost array membrane. Integr. Biol..

[18] Harris A.R., Charras G.T. (2011). Experimental validation of atomic force microscopy-based cell elasticity measurements. Nanotechnology.

[19] Alcaraz J., Buscemi L., Grabulosa M., Trepat X., Fabry B., Farré R., Navajas D. (2003). Microrheology of human lung epithelial cells measured by atomic force microscopy. Biophys. J..

[20] Kishino A., Yanagida T. (1988). Force measurements by micromanipulation of a single actin filament by glass needles. Nature.

[21] Tseng Y., Schafer B.W., Almo S.C., Wirtz D. (2002). Functional Synergy of Actin Filament Cross-linking Proteins*. J. Biol. Chem..

[22] Kang W., McNaughton R.L., Yavari F., Minary-Jolandan M., Safi A., Espinosa H.D. (2014). Microfluidic Parallel Patterning and Cellular Delivery of Molecules with a Nanofountain Probe. SLAS Technol..

[23] Kohl P., Camelliti P., Gallagher J.O., McCulloch A.D. (2006). Micropatterned cell cultures on elastic membranes as an in vitro model of myocardium. Nat. Protoc..

[24] Hayashi K. (2006). Tensile Properties and Local Stiffness of Cells. Mechanics of Biological Tissue.

[25] Roca-Cusachs P., Alcaraz J., Sunyer R., Samitier J., Farré R., Navajas D. (2008). Micropatterning of Single Endothelial Cell Shape Reveals a Tight Coupling between Nuclear Volume in G1 and Proliferation. Biophys. J..

[26] Sun Z., Martinez-Lemus L.A., Hill M.A., Meininger G.A. (2008). Extracellular matrix-specific focal adhesions in vascular smooth muscle produce mechanically active adhesion sites. Am. J. Physiol.-Cell Physiol..

[27] Satcher R.L., Dewey C.F. (1996). Theoretical estimates of mechanical properties of the endothelial cell cytoskeleton. Biophys. J..

[28] Bathe M., Heussinger C., Claessens M.M.A.E., Bausch A.R., Frey E. (2008). Cytoskeletal Bundle Mechanics. Biophys. J..

