# Supplementary material for: Study of the Effect of Cell Prestress on the Cell Membrane Penetration Behavior by Atomic Force Microscopy
Source: Micromachines (Basel). 2023 Feb 5;14(2):397. doi: 10.3390/mi14020397 (PMC9961200; doi:10.3390/mi14020397)
Supplement: Supplementary file 1 [file micromachines-14-00397-s001.zip › micromachines-2088754-supplementary.pdf]

Figure S1. The micro-groove patterns were formed by photolithography. First, the silicon substrate was spin-coated with negative photoresist (SU-8 2005, Microchem) and exposed to ultraviolet light through the glass mask with the micro-groove pattern. The mask with special micro-groove patterns protects some areas of the photoresist from irradiating by UV and exposed others to UV. After that, the unexposed photoresist was washed away during the development, leaving behind a micro-groove template. Then the mixture of 10:1 ratio of PMDS to curing agent was poured on the top of the template and cured at 60 °C for 12 h in the heater. After curing, removed it from the template and stuck it to another piece of PDMS (top piece). Then the culture dish was prepared.

Figure S2. Stretching equipment.

Figure S3. The cells grow in micro pattern with groove width of 5 μm (a), 10μm (b) and 15 μm (c)after seeding for 12 hours.

Figure S4. The representative Force-Displacement curve of AFM tip penetrating the cell.

Figure. S1

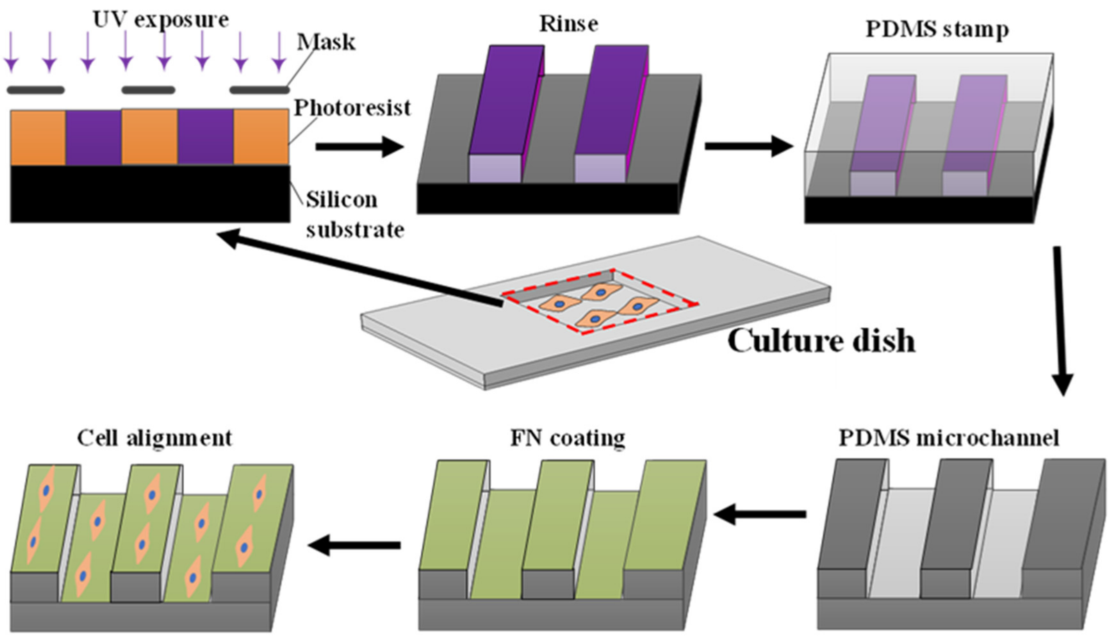
UV exposure
Mask
Photoresist
Silicon
substrate
Rinse
PDMS stamp
Culture dish
PDMS microchannel
FN coating
Cell alignment

Figure. S2

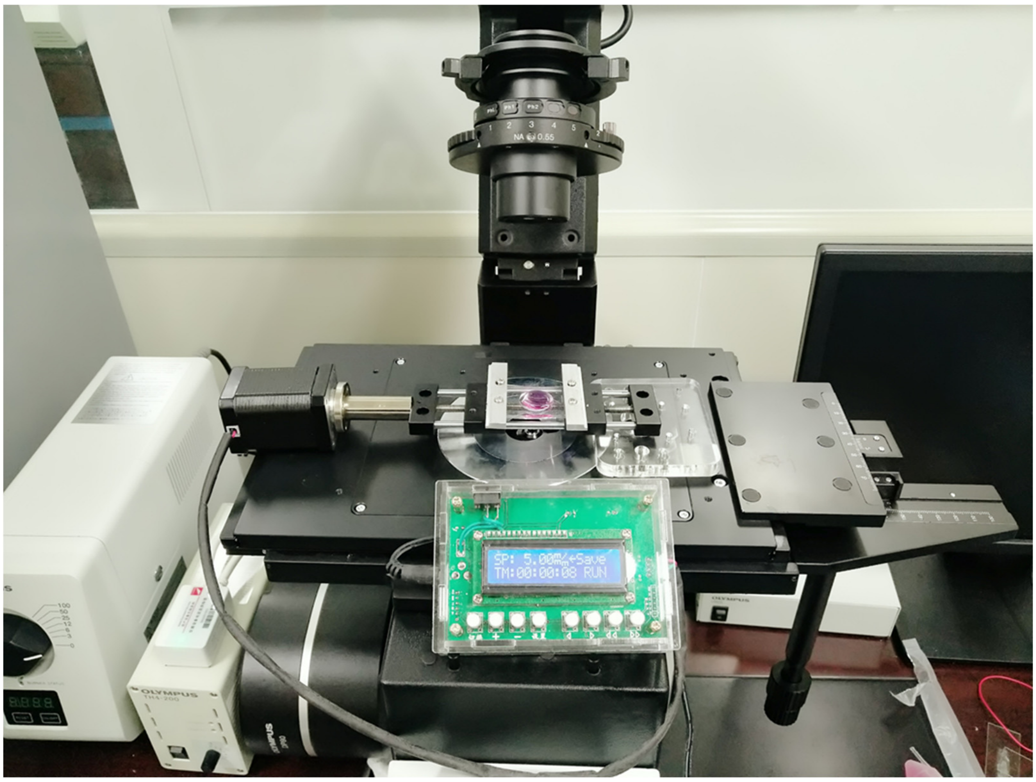

Figure. S3

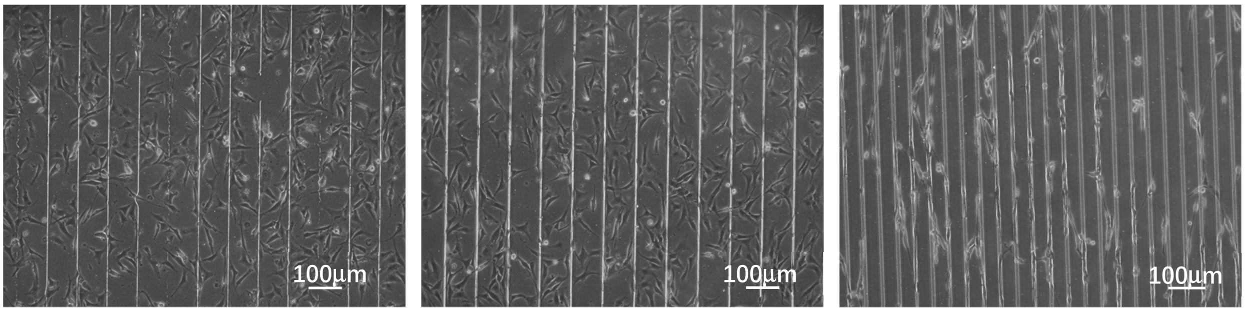

(a) (b) (c)

Figure S4

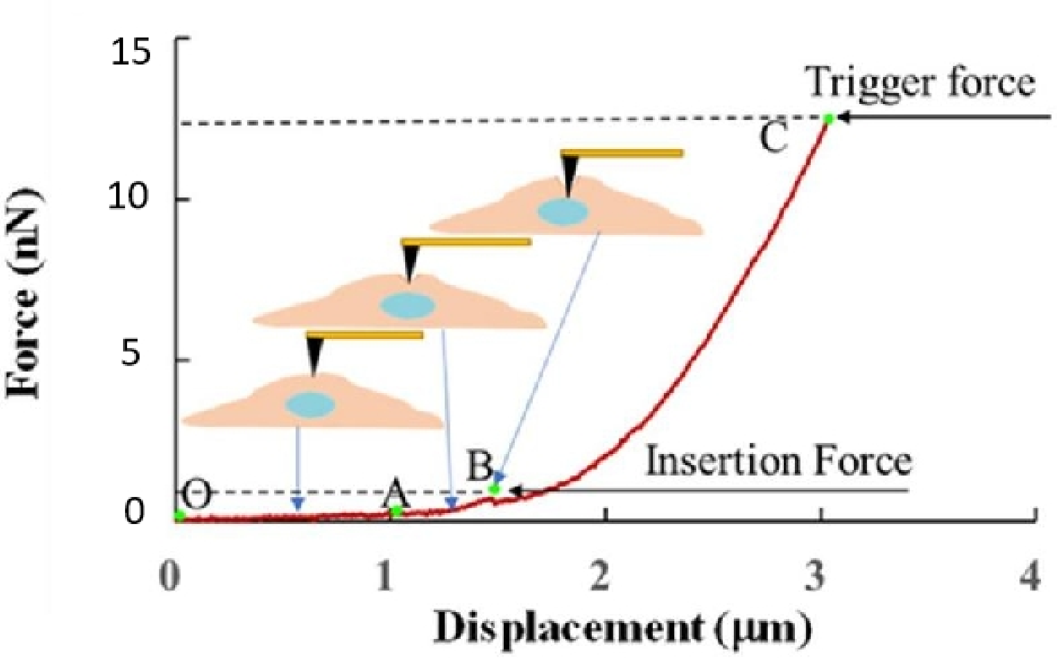

15
10
5
0
Force (nN)
Trigger force
C
B
Insertion Force
O
A
0
1
2
3
4
Displacement (µm)
